# Key findings on women’s reproductive health: the Korea Nurses’ Health Study

**DOI:** 10.4069/kjwhn.2023.06.14.1

**Published:** 2023-06-30

**Authors:** Chiyoung Cha, Heeja Jung

**Affiliations:** 1 College of Nursing, Ewha Womans University, Seoul, Korea; 2 College of Nursing, Konyang University, Daejeon, Korea

## Introduction

The Korea Nurses’ Health Study (KNHS; http://www.nhskorea.kr) is a large-scale prospective cohort study currently being conducted among female nurses in Korea. The study is based on the protocol and content of the United States Nurses’ Health Study 3 (US NHS3), adapted to account for Korea’s cultural and hospital organizational characteristics [1,2]. The purpose of the KNHS is to examine the effects of environmental, occupational, and lifestyle risk factors on the health of women of reproductive age [1]. Nurses were chosen as participants for the KNHS because their advanced understanding of disease helps ensure the validity of responses, and their commitment to study participation can help improve the follow-up rate, both of which are crucial factors in cohort studies.

The participants of KNHS are female nurses of reproductive age, ranging from 20 to 45 years old at the time of baseline data collection (the first survey). A total of 20,613 nurses from hospitals across Korea participated in the initial survey. Subsequent follow-up surveys have been conducted regularly to examine factors affecting women’s health and to track their progression into middle age. The first wave of the KNHS took place between 2013 and 2015, the second wave between 2016 and 2018, and the third wave between 2019 and 2021. The fourth wave began in 2022 and is currently in its second year. Starting with the fourth wave, the participant pool has been expanded to include nurses in their 20s. An online survey system was developed to administer the KNHS. Whenever a new survey is created, participants in the baseline data collection process are sent a text message encouraging them to participate in the follow-up surveys. The nurses voluntarily participate by using a link to the questionnaire homepage provided in the text message.

The main variables included in the first wave of the KNHS were demographics, weight, height, health behaviors, illness, medication, family history, pregnancy, mood, employment, occupational exposure, and subjective perception of current health. Follow-up surveys introduced additional variables alongside the content from the first wave. To date, a total of 11 surveys have been developed and completed as part of the KNHS, and a 12th survey is currently under development. Moreover, since 2014, pregnant nurses have had the opportunity to answer separate survey questions focusing on early pregnancy and post-pregnancy [1].

In 2016, during the first year of the second wave, 2,000 nurses who had participated in the baseline survey underwent anthropometric measurements (e.g., waist circumference), had their blood pressure evaluated, and provided blood samples and toenail clippings, which were analyzed in conjunction with the questionnaire data [3]. Additionally, for 500 of these 2,000 participants, serum anti-Müllerian hormone (AMH) levels were measured from blood samples to investigate ovarian function [4].

## Key findings on women’s reproductive health

Over 30 papers have been published using KNHS data in the fields of women’s health, physical and psychological health, health behaviors (including eating habits), and the nursing workforce. To date, eight of these papers have specifically addressed issues related to women’s health. Upon analysis of the data collected from the KNHS, the prevalence of diseases such as breast and gynecological cancers was found to be quite low. This is likely because the KNHS initially targeted relatively young women of reproductive age. In addition to gynecological cancer, several other problems related to women’s health have been identified within the KNHS, including issues pertaining to menstruation, hormone levels, postpartum health, and uterine leiomyoma.

Issues related to menstruation included the use of menstrual sanitary products, menstrual cycles, and menstrual distress. A study on the use of menstrual sanitary products was conducted in response to societal concerns about hazardous materials in these products [5]. Questionnaires were incorporated into survey 7 to identify and analyze patterns of sanitary product usage. Analysis of survey 7 data (n=8,658) revealed that the majority of participants used disposable menstrual pads (89%), and respondents expressed anxiety regarding safety issues. A study examining factors contributing to the length and regularity of menstrual cycles in 12,851 participants from survey 3 found that 21% experienced irregular menstrual cycles. Notably, frequent shift work among childless nurses was associated with irregular cycles, and women who reported prolonged standing or frequent heavy lifting during work also often experienced irregular cycles [6]. These findings align with the results of a study investigating the relationship between occupational characteristics and irregular menstrual cycles in female workers, based on data collected from the Korea National Health and Nutrition Examination Survey V (2010–2012) [7], as well as findings on the relationships of menstrual function with work schedule and physically demanding work, based on analysis of data from the US NHS3 [8]. Another study explored the associations between depressive symptoms and menstrual distress [9]. Surveys conducted between 2018 and 2019 (n=6,878) indicated that depressive symptoms increased menstrual distress in the premenstrual and menstrual phases by 1.60 times and 1.65 times, respectively. Given the evidence suggesting that improved working environment may be related to women’s reproductive health, it will be important to further investigate these effects using data from the KNHS.

Research on women’s hormone levels has focused on AMH and polycystic ovary syndrome (PCOS) in relation to health and well-being. In one study, researchers specifically analyzed data from 448 individuals who participated in surveys 3 and 5 of the KNHS and underwent AMH blood testing. The results revealed that body mass index (BMI), total cholesterol level, and low-density lipoprotein level were negatively correlated with AMH, while high-density lipoprotein level demonstrated a positive correlation with AMH [4]. These findings suggest that weight gain, BMI, and changes in lipid profile may be associated with women’s reproductive health. Consequently, it is essential for Korean women to maintain a healthy weight and manage their lipid profiles by adopting better lifestyle habits. Another study examined 11,866 participants from survey 3 and found that the prevalence of PCOS was 7.1%. Additionally, BMI was linked to menstrual irregularity, facial acne, and hirsutism in the group with PCOS [10].

Researchers conducted a comparison of postpartum depression in women from Korea and the United States [11]. Data from the KNHS (n=1,244) and the NHS3 (n=2,742) revealed that a higher percentage of Korean women exhibited clinical symptoms of postpartum depression (45.9%) compared to their United States counterparts (3.4%). However, for both groups, the presence of prior depressive symptoms and poor sleep satisfaction were identified as predictors of postpartum depression.

Two studies have been published on uterine leiomyoma. One study examined the association between weight change and uterine leiomyoma in 5,338 pregnant participants from survey 1. The results showed that 4.1% of participants (n=210) had been diagnosed with uterine leiomyoma, and weight gain after the age of 18 years was significantly correlated with an increased risk of uterine leiomyoma [12]. Another study employed a longitudinal analysis to explore the relationships of menstrual and reproductive factors with the risk of uterine leiomyoma in premenopausal women [13]. That study included 7,360 women who participated in the survey between 2014 and 2016 and underwent follow-up in 2021. The analysis utilized Cox proportional hazards models and found that age at menarche, menstrual cycle length, parity, and age at first birth were inversely associated with the risk of developing uterine leiomyoma.

## Conclusion

The KNHS is the first large-scale cohort study of working women of reproductive age in Korea. Its purpose is to investigate factors affecting the general health and reproductive health of women, an area not explored in previous studies. The data for the KNHS have been collected over 10 years, primarily from healthy women. As the participants age, the prevalence of chronic diseases and other health issues is expected to increase. By analyzing the accumulated data on lifestyles and work environment influences, valuable insights into health and disease can be uncovered. The data collected from the KNHS can contribute to the development of health indicators and health improvement policies by elucidating the causes of diseases, including gynecological cancer, in Korean women. Additionally, the work intensity of Korean nurses is much higher than in other developed countries. The nurse-to-patient ratio in intensive care units in Korea is 1:2.48 in large tertiary hospitals and 1:4.20 in hospitals with 300 to 499 beds, whereas the ratio is at least 1:1 in England [14]. Therefore, it is important to explore how the work characteristics of female nurses affect their health, and the KNHS is expected to provide important data to support legislation and policies aimed at improving nurses’ work environments.
